# Will the last be first and the first last? The role of classroom registers in cognitive skill acquisition

**DOI:** 10.1371/journal.pone.0197746

**Published:** 2018-05-29

**Authors:** Francesca Borgonovi, Maciej Jakubowski, Artur Pokropek

**Affiliations:** 1 Organisation for Economic Co-operation and Development (OECD), Directorate for Education and Skills, Paris, France; 2 University of Warsaw, Faculty of Economic Sciences, Warsaw, Poland; 3 European Commission, Joint Research Centre, Unit B4—Human Capital & Employment, Ispra, Italy; 4 Institute of Philosophy and Sociology, Polish Academy of Science, Warsaw, Poland; Universite de Bretagne Occidentale, FRANCE

## Abstract

The paper estimates the effect of students’ position in the classroom register on their academic performance. We use a unique dataset from Poland which contains information on the academic outcomes of students in the humanities, science and mathematics lower secondary school exams as well as the position students occupy in their classroom register. We find that students whose names are recorded near the end of the class list have lower performance than those students whose names are recorded near the beginning of the list. The effect appears to be larger for performance in the humanities exam, and for low-achieving boys who attend large classes.

## Introduction

There is ample evidence that classroom dynamics are important for learning [1,2] and that even small differences in classroom conditions are associated with students’ motivation to learn and the effort they invest in learning [3]. While correlational evidence abounds, causal evidence is scarce [1]. Moreover, little is known about how different groups of students respond to given conditions and how teachers’ practices interact with other classroom factors–such as class size, the average level of achievement of a student’s classroom peer composition–to shape students’ academic performance [4,5].

Schools represent one of the first environments in which students become part of a large group of peers–the class–under the lead of an adult who, at the start, knows relatively little about each member of the group. Class membership is recorded in the classroom register, where students are listed in alphabetical order based on their surname. The classroom register is a book with the function of allowing teachers to record absences and grades. Digital registers were extremely rare in Poland before 2012. However, teachers also use the classroom register in other ways: the classroom register in fact constitutes a complete record of classroom members and in Poland, like in many other countries, teachers use the register to “randomly” select students when needed for on-the-spot questions.

We use students’ placement in the classroom register to indirectly estimate if certain monitoring practices, such as on-the-spot testing, can foster students’ learning. We hypothesise that the learning benefits associated with on-the-spot tests will be stronger among students who are called more often within a class, and that these students will tend to be those who appear near the beginning of the classroom register. Students’ position in the classroom register is not correlated with students’ underlying ability, and other underlying factors that are associated with learning such as level of intrinsic motivation to learn and capacity for self-regulation. Registers constitute an easy to access list that many teachers in Poland often use for selecting students for on-the-spot tests to monitor students’ progress and give students an incentive to study throughout the school year rather than in preparation of specific written exams. While teachers use registers with the idea of randomly selecting students, unconscious heuristics in human visual processing mean that they instead follow typical and predictable paths when they scan long lists of names in the register. That provides a natural experiment that we explore in this paper.

We use confidential data from seven cohorts of students who took the lower-secondary exams in Poland between 2005 and 2011 to explore if students’ position in the classroom register is associated with differences in performance. Lower-secondary exams are compulsory for all 15-year-olds schooled in Poland and have two components: a humanities component–which includes a test of Polish language and history–and a mathematics-science component–which includes a mathematics, biology, geography and physics test.

The paper is structured as follows. First, we discuss theory and hypotheses, building our study on insights from experimental cognitive psychology on how individuals visually process lists. We then describe the data and the methods we use to empirically estimate the effect of surname initial on academic performance. Finally, we discuss results and illustrate conclusions and directions for future research.

### Theory and evidence on the use of classroom registers

Research in visual cognition identifies clear serial positioning effects that are primarily, but not exclusively, due to memory effects: the first and last items in any presentation have the highest recall. For written lists, these are items at the top and bottom, as the typical scanning behaviour for text written in European languages is left to right or top to bottom [6–9]

In Poland, classroom registers typically take the form of one-column lists displaying firs the surnames and then the first names of students. We hypothesise that teachers scan registers using typical scanning behaviour: from top to bottom and, less often, from bottom to top [10,11]. We further hypothesise that scanning behaviour will be mitigated by the fact that individuals tend to avoid extreme categories (the very first and the very last student).

Our main hypothesis is that students who are near the beginning or near the end of the classroom register will have higher performance than students who are the very first or the very last on the list and students in the middle of the list. Furthermore, because we expect that teachers more often start from the top of the list rather than from the bottom, that students near the beginning of the list will have higher performance than those near the end.

Having a position in the classroom register that reduces the frequency of being checked by teachers will be particularly problematic for those who have a greater tendency to put less effort into schooling and who are more prone to having behavioural problems or exhibiting poor classroom discipline. The capacity to exercise self-control is critical for individual success [12,13] and students who exhibit more self-control and who are more conscientious, have been shown to have better academic records than those without [14,15]. Boys are more likely to suffer from behavioural and classroom discipline problems [4,16]. Boys’ identities are often defined by a relative lack of interest in schooling [17–19] and they generally reveal lower levels of conscientiousness and self-regulation than girls [20,21]. Therefore, our second hypothesis is that the effect of students’ position in the classroom register will be stronger for boys, and will be particularly strong among lowest-performing boys who generally suffer the most from lack of self-regulation, motivation and discipline. Our final hypothesis is that the effect of students’ position in the classroom register on their performance will be particularly acute in very large classes because teachers may rely more on the external aid registries constitute to select students for on-the-spot testing in large classes. Additionally, as the length of the registry increases, differences in visual processing may become more acute. Finally, in small classes, individual students have an overall higher probability that they will be selected for on-the-spot-tests than in larger classes such that all students will have an incentive to put regular efforts in their studies. In larger classes, the overall probability that an individual student has of being called for on-the-spot-tests is lower, but disparities in such probability dependent on registry position will be higher and, as a result, differences in student effort will be larger.

## Data and methods

### Data sources and construction of research datasets

Our study is based on seven cohorts of students who sat the lower secondary school national exams in Poland between 2005 and 2011. We exclude students with special needs and students who do not meet the identifying conditions discussed below (class size between 10 and 35 students, classes with proper ID and valid classroom register identifier).

Exam results are not directly comparable between years because different sets of test items were employed in each year. Such items could, for example, vary in overall level of difficulty and composition. Therefore, in order to pool results from all cohorts, we estimated performance scores using Item Response Theory (IRT) models and standardised all results to ensure that our performance measure across years is not determined by the specificity of the test items and how students responded to such items in a specific year. For each cohort we employ a three-parameter logistic (3PL) IRT model with expected *a posteriori* (EAP) estimates of students’ abilities. The 3PL IRT model is a latent variable model that allows estimating the level of performance of individuals using their responses to different test items, whereby responses are expressed on a categorical scale. Unlike other simpler scoring methods, the IRT approach does not assume that each item is equally difficult and the 3PL specification additionally allows to estimate the degree of the association between the latent trait and test items, the level of guessing behaviors and utilize this information in the estimation of the performance indicators [22]. Scores were then standardised (μ = 100, σ = 15) separately for each cohort. In addition, as we are pooling data for different cohorts of students taking exams at different years, we included year dummies (year fixed effects) in all regressions to control for possible differences between exams in different years and for differences between student cohorts.

Our dataset uses information provided by eight regional examination boards who hold confidential student level results. Boards were asked to prepare anonymous data including student responses to the test, gender, age, whether the student suffers from dyslexia, the first letter of the student surname, identifiers of the school students attend, their class, the location of the school, whether the school is public or private, and an identifier of the regional board. From the data we also constructed the class mean intake score defined as the average of the results that students in the same class as the respondent in lower secondary school had obtained in the exam that they sat at the end of primary school (which takes place right before students enter lower secondary education. This variable reflects class compositional effect, which is usually highly predictive of individual student achievement and, crucially, is not affected by the specific circumstances that occur in classrooms in lower secondary school, because these exams pre-date lower secondary education. We also calculated school and class size as the number of students taking the final examination in lower secondary school. We use these variables as additional controls in our regressions.

We identify the order in which students are listed in the classroom register using the student identifier within the school and class and check for accuracy using surname initial. Students without a valid classroom register position identifier are not considered in the analyses. We also excluded all students in classes with less than 10 or more than 35 students as such classes are unusual and students who attend such classes may differ along unobservable characteristics from other students in our dataset. Table 1 shows sample sizes and descriptive statistics before and after deletions. Exam results across different samples are almost identical. Mean achievement is higher in the sample of large classes. This positive association reflects the fact that in Poland large schools and classes are predominantly found in large cities and in sought-after schools [23].

**Table 1:** Sample statistics.

	Humanities			Math-science		
	Mean	SD	N	Mean	SD	N
**Original data**	100	15	3 269 012	100	15	3 267 640
**Classes with proper IDs and classroom size 10–35 students and students ordered according to surnames**	100.3	14.8	2 800 276	100.3	14.9	2 799 035
**Main estimation sample: Large classes with proper IDs, students ordered according to surnames and classroom size 28–35 students**	103.8	14.4	603 595	104	15.1	603 384

To confirm our hypothesis that the underlying mechanism leading to a classroom register position effect stems from teacher’s use of classroom registers to call students for on-the-spot-testing we collected data from a sample of 150 teachers. Data were collected directly from registers to examine patterns in students’ grades and a survey distributed to teachers asking them to report how they used classroom registers.

Data on marks were collected from 91 classrooms (in five schools and three different student cohorts) on the number of marks students received in each of the subjects that were tested in the Polish exams and that were recorded in the classroom registers. Because of confidentiality reasons, the sample was non-random but rather from schools that agreed to anonymously code classroom register data with a number indicating students’ position in the register, the number of marks each student received in the first and in the second semester by subject, and the student’s final grade in the subject.

## Methods

We assume that a student position in the classroom register is orthogonal to students’ underlying abilities or factors that may influence academic performance, such as level of intrinsic motivation to learn and capacity for self-regulation. Therefore, we assume that the relationship between position and performance in exams will be exogenous. Our strategy rests on the assumption that students’ position in the classroom register is not correlated with other factors that may influence performance. One such factor could be the student surname initial. Poland is a country with few migrants, a very small Roma community, and years of egalitarianism forced by a communist regime means that surnames very rarely can provide information that can be used to discriminate–positively or negatively–different students. Surnames that are indicative of social position (for example, because they denote an aristocratic origin) are very rare in Poland and, most importantly, the few that exist do not occupy specific positions in the alphabet and therefore are not associated with the probability that students from a different social class will tend to be placed near the beginning, middle, or end of the classroom register.

While we cannot directly test the proposition that surname initials (and consequently the probability of being recorded near the beginning or near the end of the classroom register) are not correlated with important characteristics that affect learning, we run several checks to see if, for example, surname initials are correlated with performance beyond the effect they have on position in the classroom register. We do this by estimating a set of regressions of key student and school characteristics as dependent variables and regressed them on dummy variables denoting surnames initials. Results indicate that there is no association between surname initials and a student’s class average intake score, gender, whether the school students attend is public, whether the student won any national competition in a subject, whether the student is dyslexic, school size or class size.

We expect the relationship between test performance and classroom register position to be bimodal with two peaks around the beginning and the end of the list and three lower points around the very first, the very last, and the middle position. In sum, this is the combined effect of teachers picking up more often those in the beginning of the list but rarely those very first and sometimes starting to read the list from the end but very rarely asking students who are the very last on the list.

**Fig 1:**
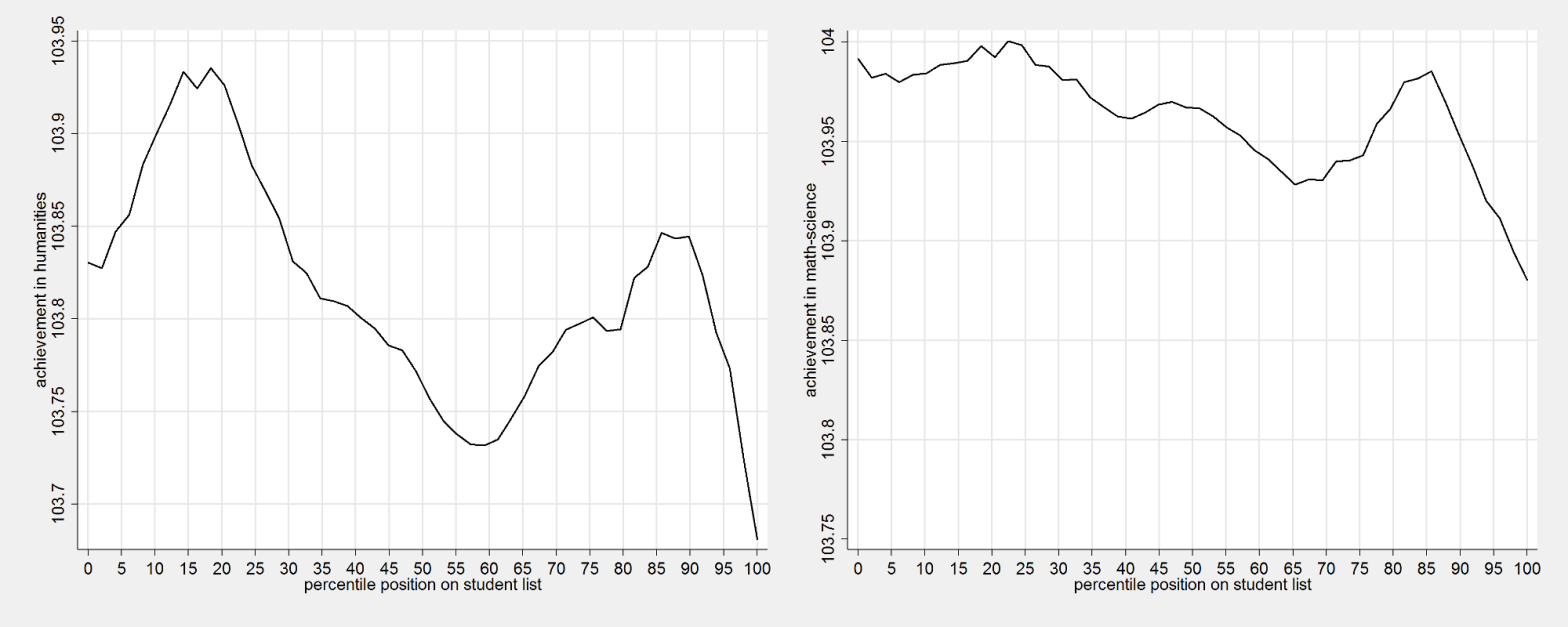
Student achievement and percentile position in classroom register. Note: Classes with 28–35 students ordered according to surnames.

Fig 1 shows a highly non-linear relationship between achievement in the humanities exam and in the mathematics-science exam and students’ position in the classroom register. Student position is displayed using percentiles to allow comparisons between classes of different sizes. The left panel (humanities) displays a bimodal distribution with a performance advantage for those students that are placed between the 15^th^ and the 20^th^ percentile in the register but also for those around 85^th^-90^th^ percentile. Students who are the very first on the register perform lower than those who immediately follow them. Performance then declines up to the middle. Another performance peak is observed for students who are around the 90^th^ percentile, while those at the end of the list perform much lower than all other students.

The right panel shows the relationship between students’ position in the classroom register and performance in the mathematics-science exam. Performance differences are generally much smaller than those observed for the humanities and three peaks can be observed. In line with the humanities exams students around the 15-25^th^ percentile have the highest performance, performance declines, peaks again at around the 45-50^th^ percentile, declines again, then peaks at around the 90^th^ percentile and performance then declines for students at the very end. The relationship between performance and students’ position in the classroom register is more noticeable in larger classes.

To flexibly model this highly non-linear relationship we employed a B-spline (basis-splines) regression model and focused on the two peaks observed across all test areas. This regression model allows high flexibility in modelling nonlinearities while providing tools to test for significance in performance differences between groups of students at different knots. Spline regression is sometimes called piecewise regression as it is based on piecewise polynomials that are used to fit a non-linear relationship with one regression model. This is an increasingly popular method, often discussed within the broader family of non-parametric regressions. Spline regression models fit separate regression lines within the regions between the knots and with these lines connected at the knots. It is a continuous function that allows to model non-linearities in relationships between variables at selected points (knots). The proper choice of knots is crucial for obtaining a well-fitting and meaningful function. Usually, knots are selected at certain quantiles or in regions where the function describing the modelled relationship is changing more rapidly. For this research, we used a B-Spline regression package available in Stata [24]. Below we describe our model and explain how the knots were selected.

Spline regression model may be written by:

y=B(x)+Wβ+e (1)

where y is outcome variable x is a variable in which non-linearity is modelled and **W** is a vector of control variables and **β** is a vector of corresponding regression coefficients (control variables are not required in spline regression but this is the case of our analysis). Function *B*(*x*) defines splines character. The B-spline model is characterized by (for detail see[25]):

$$B(x)=\sum_{i=0}^{K+n}\beta_{i}\left(\begin{array}{c} n\\ i \end{array}\right){\left(1-x\right)}^{n-i}x^{i}$$ (2)

where *n* is a degree of polynomial and *K* is a number of knots. This function allows for nonlinearity between the knots without sharp corners and does not lead to problems of collinearity for regression estimation as others spline functions do [26].

In practice, the application of B-spline functions can be reduced to adding vectors (spline-variables) to the data matrix with additional regressors in the model. Each vector is referring to values of *x* defined by two subsequent knots. The vector referring to a particular region of x contains values defined by the function *B*(*x*) while it takes value zero for other regions.

The choice of spline knots is arbitrary, but we checked that at least in our case such arbitrary choice has little impact on results. We start by visually analysing the relationships detailed in Fig 1 and compare achievement of students who occupy the estimated 0, 10^th^, 50^th^, 90^th^ and 100^th^ percentiles on a student classroom register list. When modelling splines, we used cubic splines with third-order polynomials to fit the spline regression model as this provided the best fit to the data with little loss in estimates precision. As a robustness check, we fitted results using different spline regression models and different knots (for example, 15^th^ instead of 10^th^ percentile or 85^th^ instead of 90^th^ percentile) (see S1 Appendix).

Our basic regression model employs four spline-variables that indicate the difference in performance between the estimated performance at the 10^th^ percentile and performances at the 0, 50^th^, 90^th^ and 100^th^ percentiles. The main comparison we make is between the very last students in the list (100^th^ percentile) and those who are close to the beginning (10^th^ percentile) but also between the very first students in the list (0 percentile) and those close to the beginning (10^th^ percentile). For simplicity’s sake we define these comparisons as *the first comparing to the second* (for the estimated performance difference between 0 and 10^th^ percentile) and *the last comparing to the second* (for the estimated performance difference between the 100^th^ and 10^th^ percentile).

## Results

Table 2 presents main results for two regression specifications using four different cut-offs for class-size. The left panel displays results for spline regressions without any additional control variables for classes with 20 students or more, 24 students or more, 28 students or more, and 30 students or more. The right panel displays results for the same specifications controlling for whether the school is located in an urban or rural setting, school size, class size, the year in which the assessment took place, the region in which the school is located, students’ gender, whether they are dyslexic and whether students won national or regional student competition in related subjects. These regressions have four spline variables that were defined to measure performance differences between students at the 0, 50^th^, 90^th^ and 100^th^ percentile in the classroom register and those at the 10^th^ percentile. Table 2 presents estimates for the difference in performance between students who are at the 10^th^ percentile of the classroom register and students who are the very first (0 percentile) and last (100^th^ percentile) as we focus on such comparisons. Negative estimates should be interpreted as a performance advantage for students who are at the 10^th^ percentile of the classroom register compared to the very first and the very last students in the register. Standard errors are provided in parentheses.

**Table 2:** Comparison between students second on the list and those first or last for different samples and regression models.

Class size:		Regression without additional controls				Regression with additional controls^a^			
		20+	24+	28+	30+	20+	24+	28+	30+
**Humanities**									
**the first comparing to the second**	**coef.**	-0.16***	-0.11***	-0.17***	-0.28***	-0.04	-0.04	-0.12**	-0.25***
	**SE**	(0.03)	(0.04)	(0.06)	(0.10)	(0.03)	(0.04)	(0.06)	(0.09)
**the last comparing to the second**	**coef.**	-0.28***	-0.25***	-0.29***	-0.42***	-0.13***	-0.17***	-0.25***	-0.37***
	**SE**	(0.04)	(0.05)	(0.08)	(0.12)	(0.04)	(0.05)	(0.08)	(0.12)
**Mathematics-science**									
**the first comparing to the second**	**coef.**	-0.14***	-0.08**	-0.05	-0.10	-0.02	0.00	-0.03	-0.10
	**SE**	(0.03)	(0.04)	(0.07)	(0.10)	(0.03)	(0.04)	(0.06)	(0.10)
**the last comparing to the second**	**coef.**	-0.29***	-0.26***	-0.21***	-0.27**	-0.17***	-0.19***	-0.18**	-0.27**
	**SE**	(0.04)	(0.05)	(0.08)	(0.13)	(0.04)	(0.05)	(0.08)	(0.12)

^a^The additional controls included in the regressions are: class average intake score, school location (whether the school is in a rural area, in a small town large town or city), school size, class size, year dummies for when the exam took place, regional examination board (Poland is divided into eight regional examination boards), gender, a dichotomous indicator determining whether the student suffers from dyslexia and an indicator for whether the student won national or regional student competition in related study subjects.

*** p<0.01

** p<0.05

Results presented in the left panel of Table 2 confirm our hypothesis that student at the very end of the classroom register list suffer a significant performance disadvantage compared to students at the 10^th^ percentile. Results remain statistically significant, albeit quantitatively smaller when the full set of additional controls is included in the estimates. They are stronger in large classes and in the humanities: the performance gap between the last student in the class and the second student in the class is 0.4 score points in the humanities and 0.3 score points in mathematics-science (for students in large classes).

The achievement difference between the first student (0 percentile) and those immediately following (10^th^ percentile) is much smaller than that between the second (10^th^ percentile) and the last (100^th^ percentile) and is only statistically significant in the humanities exam and for students attending large classes. For these students, the achievement gap between students who are the very first and those who are right below them in the register is around 0.25 score-points in the humanities, while it is smaller than 0.1 score points in mathematics-science (not statistically significant).

These results also hold under different specifications and sample choices. Tables in S1 Appendix illustrate how results are robust to the use of alternative approaches to the measurement of students’ position in the classroom register (measurement based on student IDs and measurement based on surname initial); sample selection based on class size and differently specified spline regression knots. Results displayed in S1 Appendix are in line with those presented in Table 2 suggesting that our findings are robust to the sets of assumptions we make.

Next, we turn to examining potential differences between boys and girls. In Table 3, we focus on a sample of boys and girls attending large classes (28 students of more) and we compare the effect of being near the top of the classroom register rather than being near the bottom or being the very first student in the register. In general, the effect of being at the bottom of the classroom register is stronger for boys than for girls, although differences are measured quite imprecisely suggesting large variation in the effect. For boys, the significant loss in achievement ranges between 0.35–0.38 in mathematics-science exam and 0.43–0.49 in the humanities exam, while it is close to zero for girls. The effect of being the very first in the register is significant only in the humanities exam and for boys.

**Table 3:** Performance comparison between the second on the list and the first and last on the list, by gender and test subject.

			humanities	mathematics-science
**boys:**	**the first comparing to the second**	**coef.**	-0.18**	0.01
		**SE**	(0.09)	(0.10)
	**the last comparing to the second**	**coef.**	-0.43***	-0.35***
		**SE**	(0.12)	(0.12)
**difference in the effect for girls:**	**the first comparing to the second**	**coef.**	0.16	-0.06
		**SE**	(0.13)	(0.13)
	**the last comparing to the second**	**coef.**	0.39**	0.38**
		**SE**	(0.16)	(0.17)

Note: Classes with 28–35 students ordered according to surnames

*** p<0.01

** p<0.05

Overall, the closer examination of the class position phenomenon displayed in Table 4 reveals that list order effects clearly affect only the performance in the humanities exam of boys who are at the bottom of the classroom register.

**Table 4:** Additional robustness checks: Regressions with classroom and letter fixed effects.

Subject	Comparison		No correction	Class FE	Class FE +letter FE	Letter FE
**Humanities**	**the first comparing to the second**	**coef.**	-0.17***	-0.14**	-0.26	-0.24**
		**SE**	(0.06)	(0.06)	(0.23)	(0.12)
	**the last comparing to the second**	**coef.**	-0.29***	-0.26***	-1.14**	-1.35***
		**SE**	(0.08)	(0.08)	(0.58)	(0.41)
**Mathematics-science**	**the first comparing to the second**	**coef.**	-0.05	-0.02	0.02	-0.15
		**SE**	(0.07)	(0.07)	(0.23)	(0.13)
	**the first comparing to the second**	**coef.**	-0.21***	-0.17**	-0.54	-0.97**
		**SE**	(0.08)	(0.08)	(0.60)	(0.44)

Note: FE means fixed effects (dummy variables) denoting classrooms and/or the same initial letters in student surnames.

*** p<0.01

** p<0.05

**Fig 2:**
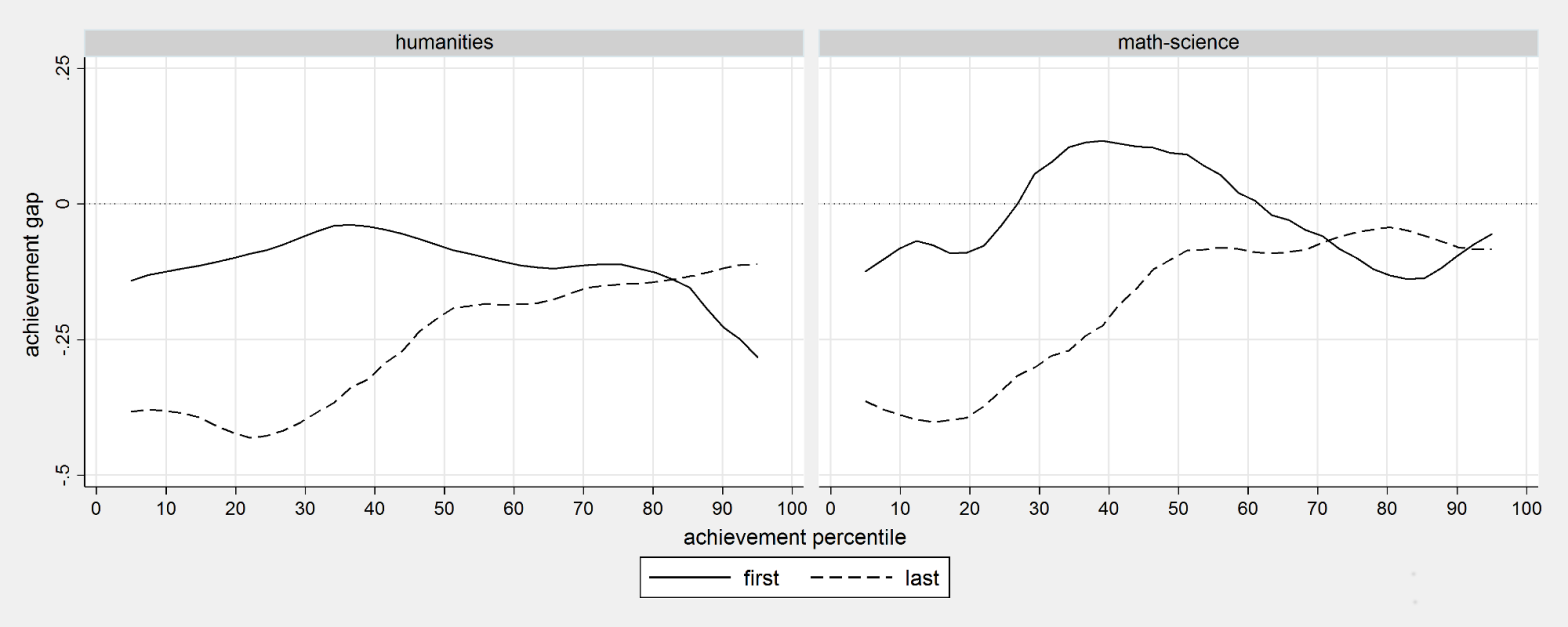
The effect of being the last and the first in the class by performance level and test domain. Note: Classes with 28 students or larger, smoothed quantile effects.

Fig 2 shows the performance gap between students who are at the top of the classroom register (solid line) and those who are at the bottom (dashed line). Students who are near the top but are not the very first as the reference category. The left panel illustrates, for students at different levels of performance, performance gaps in the humanities exam while the right panel illustrates performance gaps for the mathematics-science exam. Results were estimated using quantile regression with splines defined in the same way they were defined in previous linear regression models. Quantile regressions were developed for percentile 5, 10, 15, 20, 25 etc…of performance. Estimated coefficients were smoothed in the graph to obtain a clearer picture. Fig 2 indicates how the effect of being the last on the list differs for low and high performing students in the two exam subjects. The performance disadvantage due to being the last in the classroom register, rather than being the second, is close to 0.5 score points among the lowest achieving students but is only around 0.1 score point or not statistically significant from zero among the highest achieving students. This is true both in humanities and in mathematics-science. The effect of being the very first on the list is close to zero.

**Fig 3:**
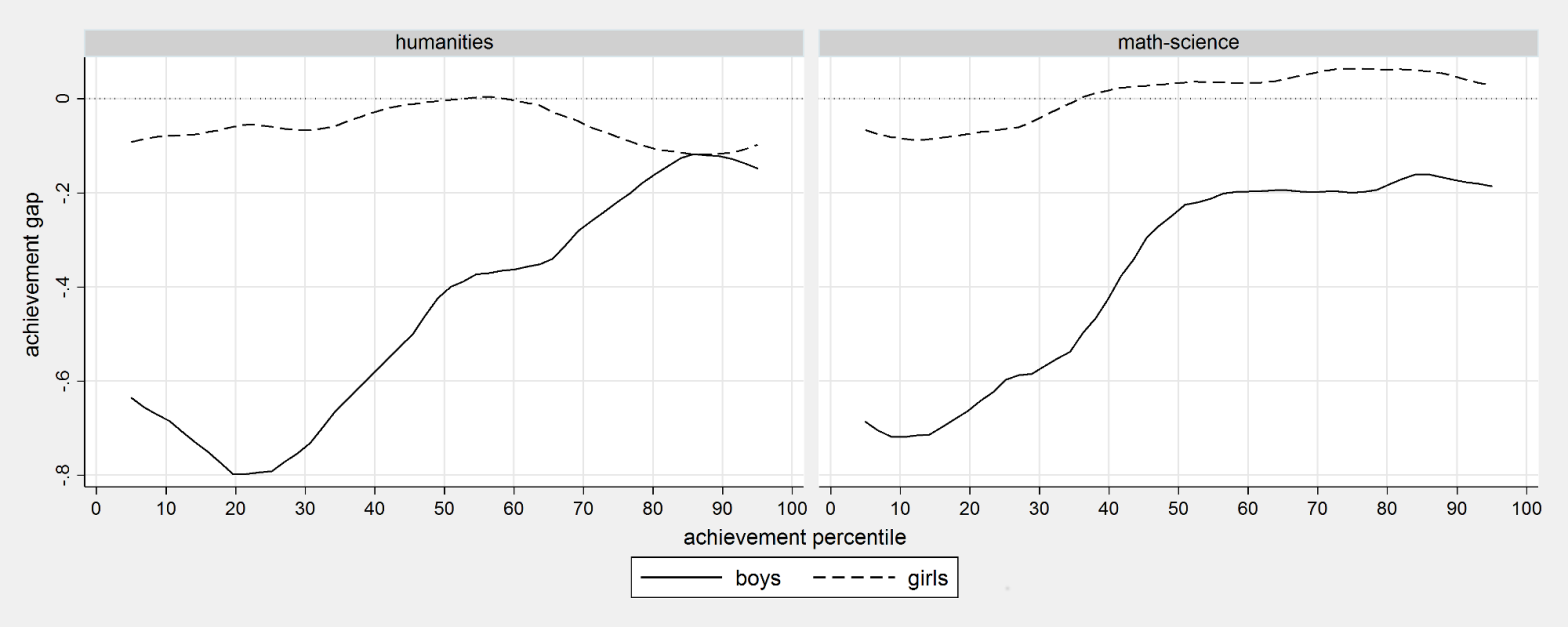
The effect of being the last in the class by gender, performance level and test domain. Note: Classes with 28 students or larger, smoothed quantile effects.

Fig 3 shows the effect of being the last in the classroom register rather than the second, separately for boys and girls at different levels of achievement. The performance disadvantage is largest among lowest achieving boys (up to 0.8 score points) and is quantitatively large even for boys with average levels of achievement (around 0.3–0.4 score points). Fig 3 indicates that girls do not suffer any clear disadvantage when they occupy a position at the bottom of the classroom register irrespective of the achievement level. This evidence indicates that at the margin, students’ position in the classroom register affects boys, and poorly achieving boys in particular, but is quantitatively negligible for girls.

**Fig 4:**
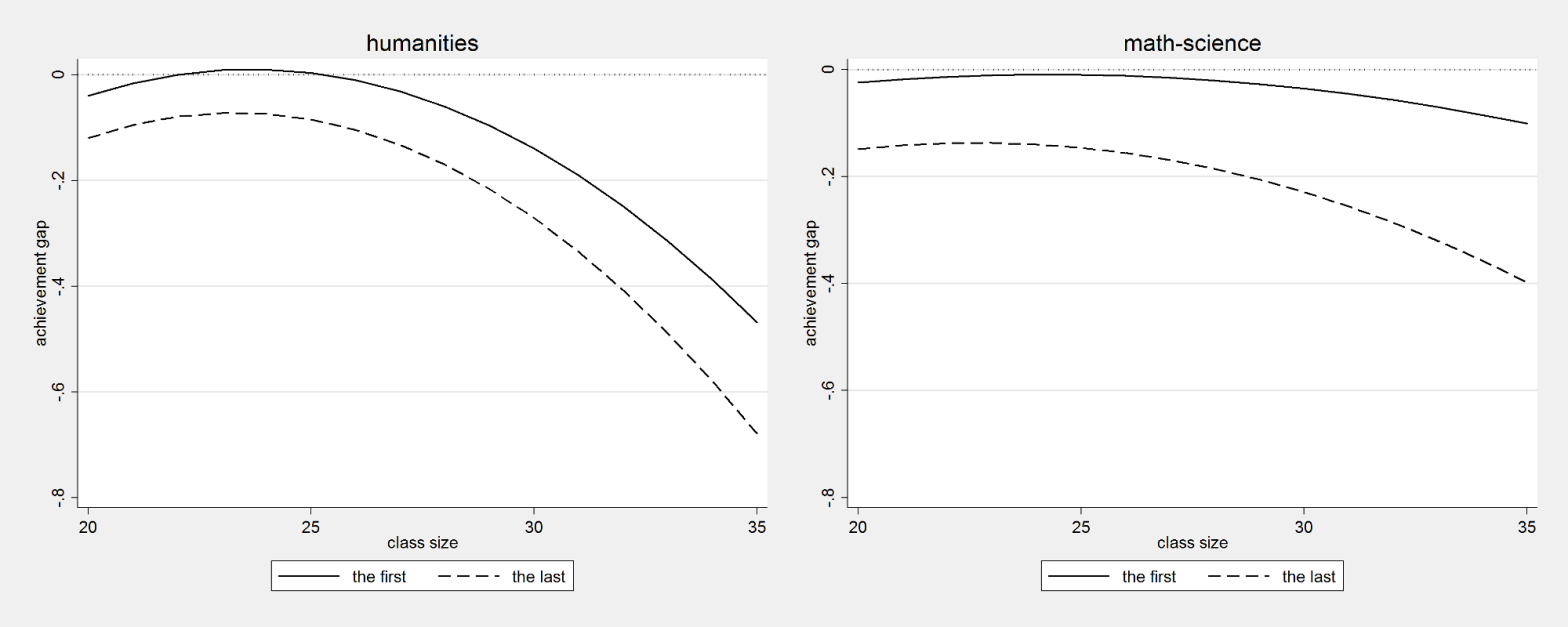
The effect of being the first and the last, by class size and test domain.

**Fig 5:**
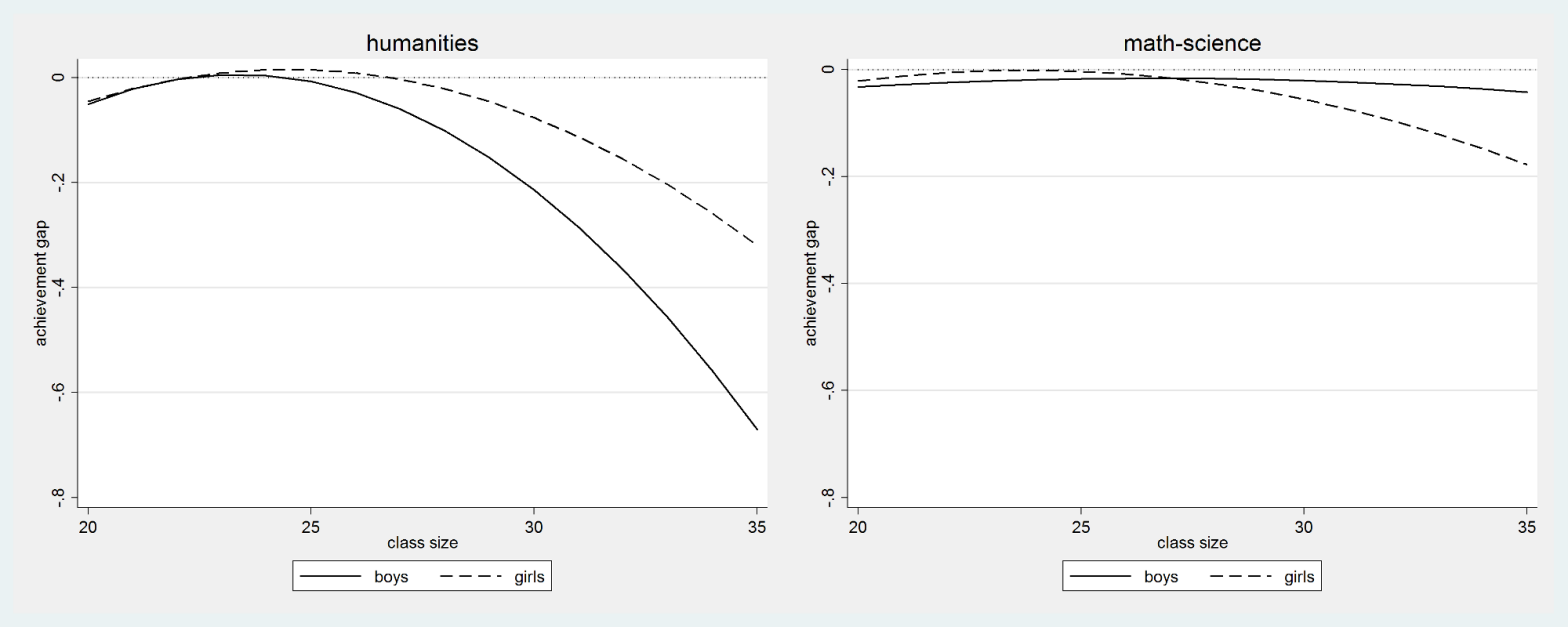
The effect of being the last, by class size, gender and test domain.

**Fig 6:**
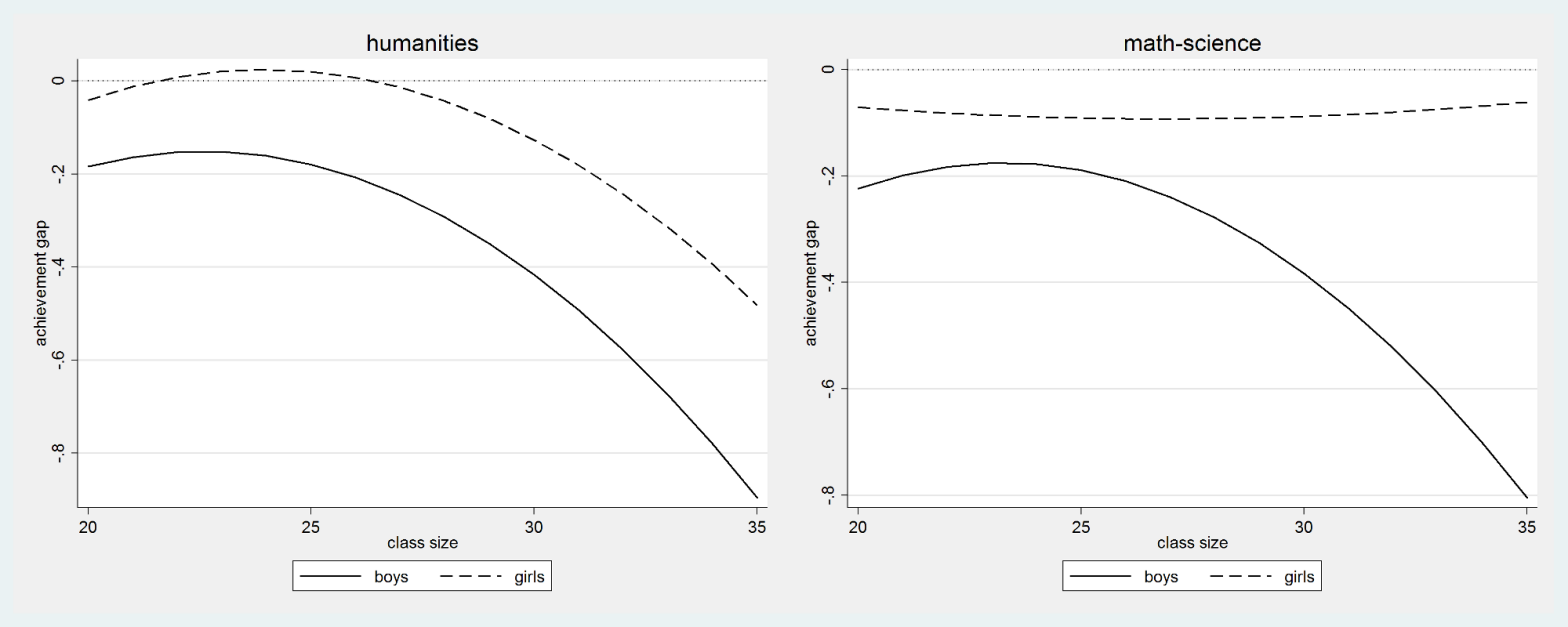
The effect of being the first, by class size, gender and test domain.

Another form of heterogeneous treatment effects in the relationship between student position in the classroom register and academic achievement comes from the nature of the class, rather than how different students will react to occupying a favourable vs. unfavourable position in the classroom register. To test the hypothesis that classroom register position is more strongly associated with performance in large classes we fitted spline regression models controlling for class size and interacting class size with regression splines. To allow non-linearity in this interaction we added squared terms of class size and interacted the quadratic term with all splines. We graphically illustrate the results in Figs 4–6.

Fig 4 confirms our hypothesis and indicates that being the very first and being the very last in the classroom register has a stronger negative effect on performance in larger classes. In the humanities exam, in classes of average size (around 24–25 students), these effects are close to zero, while in classes of 34–35 students the performance penalty approaches 0.6 score points for students who are the last in the register and 0.4 for students who are at the very first in the register. In mathematics, the effects are weaker: the effect of being the first is not statistically significant even in the largest classes, while the negative effect of being the last becomes moderate in quantitative terms in the largest classes.

In Figs 5 and 6, we graphically illustrate findings from more fine-grained analyses examining gender differences in whether the impact of being the first or the last in the class on academic performance depends on class size. Fig 5 reports results by gender of the effect of being the last, while Fig 6 reports results by gender of the effect of being the first. The effect of being the last is generally much stronger than the effect of being the first and is particularly strong with respect to performance in the humanities exam. Fig 5 shows that the effect of being the last is around 0.8 score points for boys and 0.5 score points for girls in the largest classes, while in average sized classes it is negative only for boys and small in magnitude (at around 0.2 score points). For mathematics-science the effect is negative only for boys and only for boys it follows a very similar pattern to the one observed in the humanities: the performance gap is 0.2 for average sized classes and it increases to 0.8 for boys who are the last in the classroom register in the largest classes. Fig 6 shows that in the humanities, the effect of being the first in the classroom register increases in magnitude as classes grow larger. In the largest classes, those with 34–35 students, the achievement gap is twice as large among boys as it is among girls: the effect size of being first in a very large class is 0.7 for boys and 0.3 for girls. In mathematics, the effects are negligible for both boys and girls.

We ran several robustness checks exploiting variance only within classrooms, only within groups of students with the same surnames initials, and variation among students who share the same classroom and the same surname initial but occupy different positions in the classrooms register because of the random surname composition of their classes. Looking at the within-classroom variance and the variance within students who share the same surname initial allows us to exclude biases that may arise from teachers’ using the classroom registers differently and any systematic differences in the way teachers treat students with a different surname initial.

The same spline regression model was applied in all cases but with additional fixed effects for lower-secondary classrooms, for all students with the same surname initial, and with fixed effects for both classrooms and initials. Table 4 presents results for classes of 28 students or more and main results to facilitate comparisons.

Regression estimates obtained using classroom fixed effects are very similar to those obtained when estimating the overall effect of student position in the classroom register on achievement. Table 4 illustrates that results obtained using only within-classroom variance or obtained when comparing students who have the same surname are stronger, especially for students who occupy the last position in the classroom register. However, results presented in Table 4 exploit only a very minimal source of variation so standard errors are relatively large with most of the results insignificant at the 5% level. Overall, all the significant effects are negative supporting earlier findings that being the very first compared to being second or being the very last is negatively associated with performance.

These results suggest that teachers’ practices are unrelated to classroom characteristics or surname initials other than students’ position in the register. Thus, we conclude that the results based on the whole sample without additional restrictions are a valid source of quasi-experimental information about the effect of teacher pressure on student achievement.

**Fig 7:**
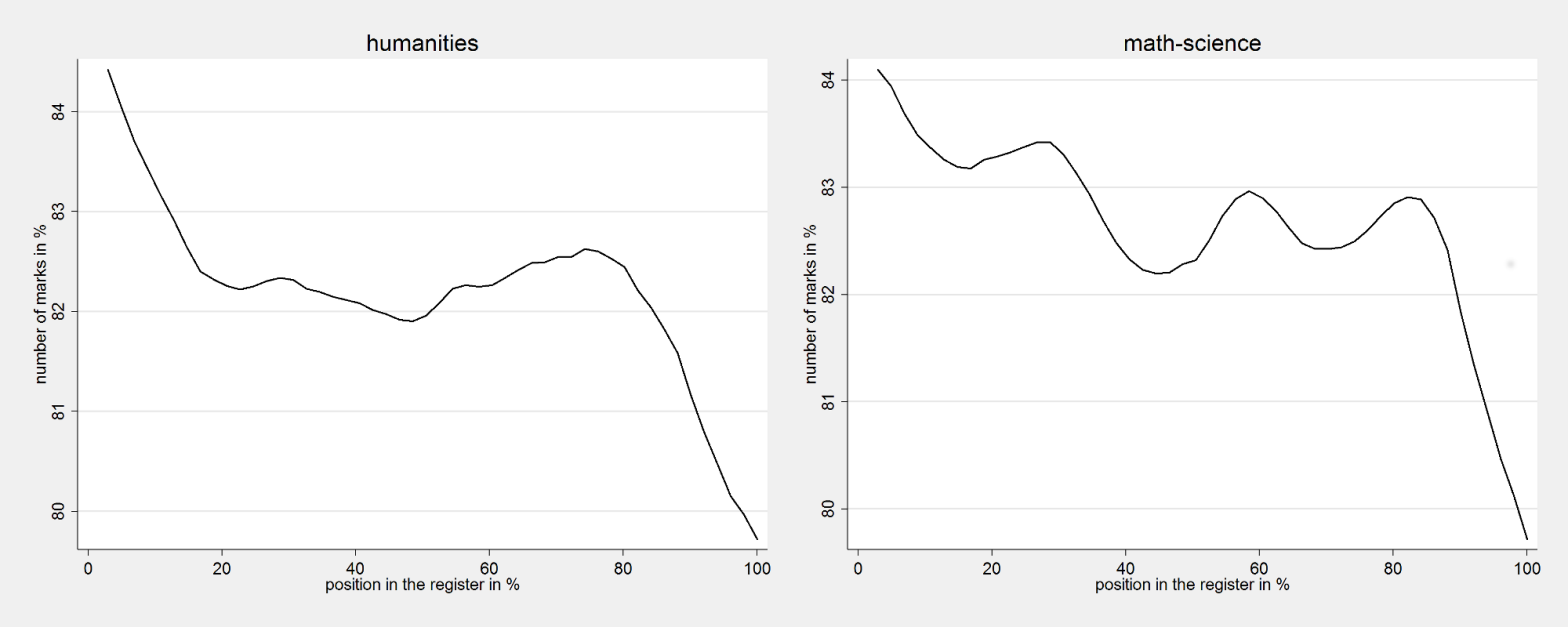
Number of marks (as a % of maximum number of marks received in a class) and student position in the classroom register.

To understand if these results are due to teachers’ use registers and how such use is linked to on-the-spot-testing, we conducted a survey of 150 teachers, 112 of whom teach in lower-secondary schools the same subjects that are tested on the Polish national examination. Fig 7 shows data the correlation between the number of marks students had at the end of the first and second semester in 2012 and the position they occupy in the classroom register in the sample of schools and classes that took part in our survey. Register position represents the percentage-rank on the list to allow comparisons of classes with different sizes. Similarly, the number of marks represents the percentage of the highest number of marks in the classroom since some teachers give more marks than others. The left panel presents results pooling students’ marks in all the subjects that are tested in the mathematics-science component of the Polish lower-secondary school exam (to aid comparability with the main analyses discussed in the paper) and on the right results for the humanities component. Fig 7 supports the hypotheses that the probability that any one student will be called for an oral examination diminishes for students who occupy positions at the bottom of the classroom register.

Twenty-seven percent of teachers reported using registers in every lesson to select students, 35% reported using the register in most of lessons, 31% reported using it rarely and 7% said that they never use it. Among those who use classroom registers in every lesson, the average time teachers reported spending on on-the-spot tests was 9 minutes and 10 minutes among those who rely on registers in most classes. Teachers who rarely use the register also reported that on average on-the-spot tests in their classes last around 6.5 minutes. The average time teachers reported investing asking students in on-the-spot tests was 1 minute larger among teachers of humanities subjects than among mathematics or science teachers.

Based on these data, we calculated a lower-bound-estimate of the average time teachers spend using registers to test students through on-the-spot checks. This amounts to 4.6 minutes per-class-lesson (on average), under the assumption that teachers who report using classroom registers in most of classes use the register in around half of their classes and teachers who report using registers rarely use the register only once every ten lessons. In Poland all class periods last 45 minutes, hence we estimate that, on average, around 10% of teacher time in the classroom is spent on on-the-spot tests of students based on the use of classroom registers.

We can use this information to derive estimates of how large classroom register position effects are: rough estimate of the difference in teacher’s time devoted to on-the-spot tests between students who are the last in the register to those who are “the second” is between 2.5% and 3.8% of a Standard Deviation (see Fig 7, the number of marks is around 82/83 for the second and around 80 for the last). This can be related to an estimated achievement difference of around 0.3–0.4 score points in humanities and 0.2–0.3 score points in mathematics-science (Table 3, classes with 28 or more students). The achievement gap is as high as 0.8 for low-performing boys.

This means that a 10% increase in teacher’s on-the-spot testing is associated with an average score increase of between 0.5 and 1.6 points (between one-thirtieth and one-tenth of a standard deviation), while for low-performing boys the increase is as high as 3 score points (or 1/5^th^ of a standard deviation). Therefore, these back-of-the-envelope calculations suggest that a 10% increase in the time teachers invest in on-the-spot testing that is devoted to an average student may be associated with a performance increase of between 4 to 11% of a standard deviation. For low-performing boys a 10% increase in on-the-spot testing may be associated with a performance increase of 20% of a standard deviation. This is a considerable difference because teachers report spending on average only around 10% of teaching time on on-the-spot testing.

## Conclusions

We compare the performance of students who are at different positions in the classroom register, a fact that is solely determined by the alphabetical ordering of lists and the surname composition of different classes. As teachers employ student position in the register for on-the-spot testing, and such position is uncorrelated with underlying ability, we can indirectly measure the effect of teacher monitoring practices on performance.

Our results indicate that students who are at the bottom of the classroom register are particularly disadvantaged, and that the performance disadvantage linked to classroom position is larger in the humanities exam (the subject where on-the-spot oral assessments are used more often) and for low-achieving boys attending large classes. Findings confirm our hypotheses of heterogeneous treatment effects of student position in the classroom register on performance according to exam subject, gender and class size. The difference in the size of the effect across study subjects suggests that the mechanism through which position determines performance is by altering how frequently students at the top and at the bottom of the register are questioned by teachers and in turn how they respond adjusting their study effort and patterns. Moreover, undertaking a test is a powerful learning experience [27,28] and students who are called more often are expected to achieve higher standards that those who are less exposed to testing. Our results are an indication of decreasing marginal returns to teacher attention and testing: when classes are large, teacher attention is dispersed over a greater number of students so that, in large classes, each individual student will be questioned less often by teacher than in smaller classes. The marginal attention students receive because they hold a favourable position in the classroom register matters more for students who are in classes where students receive comparatively low levels of attention.

Boys at the lowest proficiency levels who are at the bottom of the classroom register are at a particular disadvantage as they are the ones that gain the most from being carefully monitored by their teachers. Lack of attention and monitoring penalises poor achieving boys in particular [29], because they are the ones that are most likely to see their learning impeded by poor discipline in class, lack of attention during lectures, lack of motivation and less positive attitudes towards learning.[30–32] As boys may be less likely to invest in other forms of autonomous learning, they may particularly benefit from the learning experience that occurs from undergoing testing situations.

## Supporting information

S1 Appendix (DOCX)
