# Supplementary material for: Will the last be first and the first last? The role of classroom registers in cognitive skill acquisition
Source: PLoS One. 2018 May 29;13(5):e0197746. doi: 10.1371/journal.pone.0197746 (PMC5973596; doi:10.1371/journal.pone.0197746)
Supplement: S1 Appendix — (DOCX) [file pone.0197746.s001.docx]

**S1 Appendix**

Results displayed in Tables S1 and S2 suggest that our main findings are robust to the sets of assumptions we make to construct indicators of student position, to different regression models and to the use of different restrictions when constructing the student sample.

**Table S1. Results for differently defined classroom register’s position and for different class sizes.**

|  | Original register position | | | | Corrected register position | | | | Position based on surname initial alone | | | |
| --- | --- | --- | --- | --- | --- | --- | --- | --- | --- | --- | --- | --- |
| Class size: | 20+ | 24+ | 28+ | 30+ | 20+ | 24+ | 28+ | 30+ | 20+ | 24+ | 28+ | 30+ |
| Humanities | | | | | | | | | | | | |
| the first comparing to the second | -0.16 | -0.11 | -0.16 | -0.26 | -0.15 | -0.11 | -0.16 | -0.26 | -0.16 | -0.12 | -0.17 | -0.25 |
| the last comparing to the second | -0.28 | -0.25 | -0.27 | -0.43 | -0.27 | -0.24 | -0.27 | -0.42 | -0.23 | -0.21 | -0.27 | -0.44 |
| mathematics-science | | | | | | | | | | | | |
| the first comparing to the second | -0.14 | -0.10 | -0.04 | -0.09 | -0.15 | -0.10 | -0.04 | -0.09 | -0.16 | -0.15 | -0.12 | -0.18 |
| the last comparing to the second | -0.29 | -0.25 | -0.17 | -0.27 | -0.27 | -0.24 | -0.17 | -0.26 | -0.27 | -0.26 | -0.20 | -0.35 |

Table S2 provides results for differently specified spline regression knots (0, 15^th^, 50^th^, 85^th^ and 100^th^ percentiles instead of 0, 10^th^, 50^th^, 90^th^ and 100^th^ percentile as used in the main paper and Table S1.

**Table S2. Results obtained with differently defined spline regression knots.**

|  | Original register position | | | | Corrected register position | | | | Position based on surname initial | | | |
| --- | --- | --- | --- | --- | --- | --- | --- | --- | --- | --- | --- | --- |
| Class size: | 20+ | 24+ | 28+ | 30+ | 20+ | 24+ | 28+ | 30+ | 20+ | 24+ | 28+ | 30+ |
| Humanities | | | | | | | | | | | | |
| the first comparing to the second | -0.18 | -0.12 | -0.17 | -0.30 | -0.17 | -0.12 | -0.17 | -0.30 | -0.18 | -0.13 | -0.18 | -0.28 |
| the last comparing to the second | -0.30 | -0.26 | -0.28 | -0.46 | -0.29 | -0.26 | -0.28 | -0.46 | -0.26 | -0.23 | -0.28 | -0.46 |
| mathematics-science | | | | | | | | | | | | |
| the first comparing to the second | -0.17 | -0.12 | -0.04 | -0.10 | -0.18 | -0.11 | -0.04 | -0.10 | -0.19 | -0.17 | -0.14 | -0.21 |
| the last comparing to the second | -0.31 | -0.26 | -0.17 | -0.28 | -0.30 | -0.26 | -0.17 | -0.28 | -0.29 | -0.28 | -0.21 | -0.38 |

Table S3 illustrates detailed estimated coefficients for the spline regression model that is at the basis of the results presented in Table 3 on the performance in the humanities and in the mathematics-science exams for students attending classes with 28 students or more. The results are presented for all coefficients for control variables included in the model.

**Table S3. Full results for spline regression on performance in the humanities and mathematics-science exam of students in classes with 28 students or more.**

|  | **Humanities** | | **Mathematics-science** | |
| --- | --- | --- | --- | --- |
|  | Coef. | Std. Err. | Coef. | Std. Err. |
| **Spline dummies (baseline=10th percentile of position)** | | | | |
| Performance at 0 comparing to 10^th^ | -.116 | .063 | -.022 | .065 |
| Performance at 50^th^ comparing to 10^th^ | -.185 | .051 | -.077 | .053 |
| Performance at 90^th^ comparing to 10^th^ | -.123 | .043 | -.078 | .045 |
| Performance at 100^th^ comparing to 10^th^ | -.247 | .079 | -.178 | .082 |
| **average intake score** | .784 | .002 | .932 | .003 |
| **School location (baseline=rural)** | | | | |
| Small Town | .473 | .056 | -.236 | .059 |
| Large Town | .903 | .056 | -.010 | .058 |
| City | 1.54 | .058 | .492 | .060 |
| **Public school** | -.641 | .210 | -.010 | .218 |
| **Student is a girl** | 4.830 | .032 | -.946 | .034 |
| **Winner olympics humanities** | 26.954 | .274 | 10.639 | .284 |
| **Winner olympics math-science** | 9.100 | .196 | 27.614 | .204 |
| **Whether student is dislexic** | -.188 | .054 | -.613 | .056 |
| **Regional board ID (baseline=1)** | | | | |
| 2 | 1.759 | .074 | -.391 | .077 |
| 3 | 2.395 | .060 | -.081 | .062 |
| 4 | -.116 | .0790 | -.443 | .082 |
| 5 | .732 | .067 | -.048 | .070 |
| 6 | 1.39 | .067 | .274 | .069 |
| 7 | 2.231 | .067 | .804 | .070 |
| 8 | -.409 | .070 | -1.900 | .073 |
| **Exam year (baseline=2005)** | | | | |
| 2006 | -.267 | .0516 | -.233 | .054 |
| 2007 | -.204 | .054 | -.453 | .056 |
| 2008 | -.369 | .057 | -.156 | .059 |
| 2009 | -.229 | .059 | -.219 | .062 |
| 2010 | -.164 | .062 | -.178 | .064 |
| 2011 | .123 | .067 | -.400 | .069 |
| **School size** | -.0003 | .0003 | -.002 | .0003 |
| **Class size** | .075 | .010 | .079 | .011 |
| **Constant** | 16.670 | .419 | 5.973 | .435 |
